# Characterization of V1V2-specific antibodies present in broadly neutralizing plasma isolated from HIV-1 infected individuals

**DOI:** 10.1186/1742-4690-9-S2-O30

**Published:** 2012-09-13

**Authors:** C Krachmarov, K Revesz, R Prattipati, C Reichman, Z Lai, W Honnen, B Li, C Derdeyn, A Pinter

**Affiliations:** 1 Public Health Research Institute/UMDNJ, Newark, NJ, USA; 2 Emory Vaccine Center, Atlanta, GA, USA

## Background

Recent studies of antibodies in human plasma from infected and immunized individuals revealed an important role of the V1V2 region of the gp120 protein in immune response. The function of V1V2-specific antibodies and their potential in blocking the HIV-1 infection are still not well established. In this study we present data about the appearance of such antibodies in plasma from hundreds of patients from North America and Africa, about their clade specificity, development and neutralization activity.

## Methods

Human plasma was isolated from infected individuals and screened for anti V1V2 ELISA binding activity with gp70/V1V2 fusion glycoproteins, representing clades B, C and A/E sequences. All samples were tested in virus neutralization assay for activity versus panel B, panel C, and other Tier 2, pseudoviruses. Immunoaffinity chromatography of selected plasma, on gp70/V1V2 protein columns, was used to isolate V1V2-specific antibodies.

## Results

Most of the HIV-1 infected subjects (above 80%) have robust levels of V1V2 binding activity versus the three antigens. This activity was commonly detected in chronic HIV-1 infection. Interestingly, the development of V1V2-reactive antibodies tracked with the development of autologous neutralizing antibodies. Immunoaffinity purified V1V2-specific antibodies from selected broadly neutralizing plasma samples possessed broad neutralization activity with IC50’s generally in the 1 -20 μg/ml range.

## Conclusion

Highly cross-reactive V1V2-specific Abs are present in almost all broadly neutralizing human plasmas. These antibodies are in large amounts and can be linear, conformational or quaternary epitope dependent. Such antibodies are induced in humans (Thailand trial) and because of that the region may be considered as immunogenic. In addition, isolated anti-V1V2 antibodies show neutralization activity toward Tier 2 viruses. The above characteristics make the V1V2 region an important target for candidate HIV-1 vaccines.

